# Supplementary material for: The contribution of inflammatory astrocytes to BBB impairments in a brain-chip model of Parkinson’s disease
Source: Nat Commun. 2023 Jun 20;14:3651. doi: 10.1038/s41467-023-39038-8 (PMC10282096; doi:10.1038/s41467-023-39038-8)
Supplement: Supplementary file 1 — Supplementary Information [file 41467_2023_39038_MOESM1_ESM.pdf]

SUPPLEMENTARY MATERIAL

The contribution of inflammatory astrocytes to BBB impairments in a brain-chip model of Parkinson's disease

A. de Rus Jacquet[1,2,3,#*], M. Alpaugh[1,2,9,#], H.L. Denis[1,2], J.L. Tancredi[3,4], M. Boutin[1], J. Decaestecker[5], C. Beauparlant[5], L. Herrmann[5], M. Saint-Pierre[1], M. Parent[6,2], A. Droit[5], S. Breton[7,8], F. Cicchetti[1,2*]

[#]Contributed equally

[1] Centre de Recherche du CHU de Québec - Université Laval, Axe Neurosciences, Québec, QC, Canada

[2] Département de Psychiatrie & Neurosciences, Université Laval, Québec, QC, Canada

[3] Janelia Research Campus, Howard Hughes Medical Institute, Ashburn, VA, 20147

[4] Cell Biology R&D, Thermo Fisher Scientific, Frederick, MD 21704

[5] Centre de Recherche du CHU de Québec - Université Laval, Axe Endocrinologie et Néphrologie, Québec, QC, Canada

[6] CERVO Brain Research Center, Québec, QC, Canada

[7] Centre de Recherche du CHU de Québec - Université Laval, Axe Reproduction, santé de la mère et de l'enfant

[8] Centre de recherche en reproduction, développement et santé intergénérationnelle, Université Laval, Québec, QC, Canada

[9] Department of Molecular and Cellular Biology, University of Guelph, Guelph, ON, Canada

*Corresponding authors:
Aurelie de Rus Jacquet, PhD
Centre de Recherche du CHU de Québec - Université Laval
Axe Neurosciences
2705, Boulevard Laurier
Québec, QC, G1V 4G2, Canada
Email: aurelie.jacquet@crchudequebec.ulaval.ca

Francesca Cicchetti, PhD
Centre de Recherche du CHU de Québec - Université Laval
Axe Neurosciences T2-07
2705, Boulevard Laurier
Québec, QC, G1V 4G2, Canada
Email: francesca.cicchetti@crchudequebec.ulaval.ca

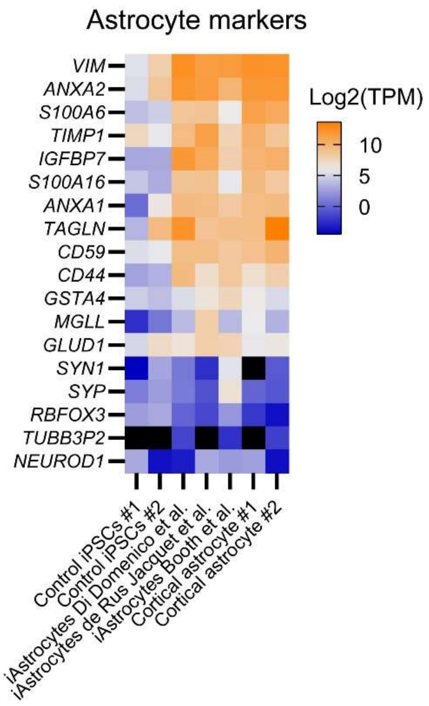

Supplementary Figure 1. Validation of the astrocyte signature across the three RNA-sequencing datasets. Heatmap showing expression levels of astrocyte and neuronal markers in all datasets used for the RNA-sequencing (RNA-seq) analysis. For comparison, the data also includes expression levels in iPSCs and human primary cortical astrocytes provided with these datasets. Abbreviation: TPM, transcripts per million. Source data are provided as a Source Data file.

A

Classification	Pathway	SIZE	ES	NES	NOM p-val	FDR q-val
Angiogenesis-related	LU_TUMOR_ENDOTHELIAL_MARKERS_UP	22	0.68	1.72	0.00	0.12
Inflammation-related	BIOCARTA_STEM_PATHWAY	14	0.75	1.70	0.00	0.13
Inflammation-related	REACTOME_DEFENSINS	47	0.53	1.55	0.01	0.33
Inflammation-related	NUMATA_CSF3_SIGNALING_VIA_STAT3	22	0.61	1.52	0.03	0.35
Inflammation-related	WALLACE_JAK2_TARGETS_UP	23	0.60	1.51	0.02	0.35
Inflammation-related	BIOCARTA_LAIR_PATHWAY	16	0.64	1.49	0.05	0.35
Angiogenesis-related	VART_KSHV_INFECTION_ANGIOGENIC_MARKERS	242	0.40	1.48	0.00	0.37
Inflammation-related	BIOCARTA_IL17_PATHWAY	14	0.65	1.47	0.05	0.37
MEK/ERK-related	BIOCARTA_BARR_MAPK_PATHWAY	12	0.66	1.44	0.09	0.41
Angiogenesis-related	REACTOME_ENOS_ACTIVATION_AND_REGULATION	20	0.58	1.43	0.05	0.41
Angiogenesis-related	REACTOME_PLATELET_HOMEOSTASIS	74	0.45	1.43	0.02	0.41
Inflammation-related	PID_INTEGRIN2_PATHWAY	28	0.52	1.40	0.06	0.44
Inflammation-related	BIOCARTA_IL3_PATHWAY	15	0.61	1.38	0.09	0.47
Inflammation-related	HADDAD_T_LYMPHOCYTE_AND_NK_PROGENITOR_UP	73	0.43	1.37	0.04	0.49
Inflammation-related	BIOCARTA_EPONFKB_PATHWAY	11	0.63	1.33	0.14	0.53

B

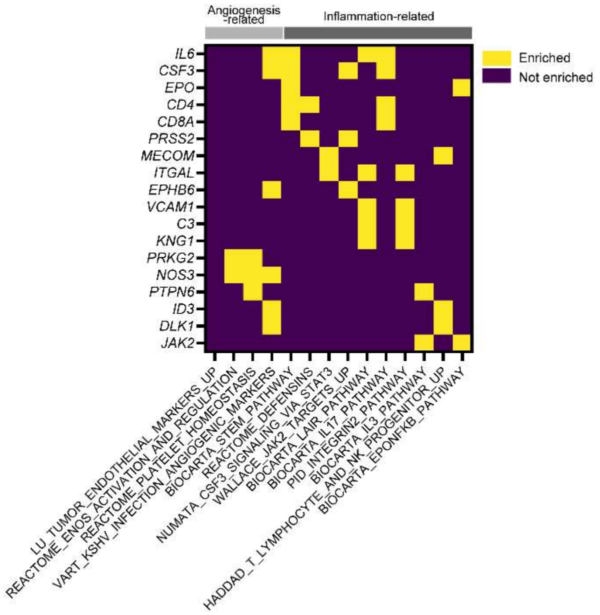

Supplementary Figure 2. Gene set enrichment analysis (GSEA) of LRRK2 G2019S vs. control iPSC-derived astrocyte RNA-seq datasets. (A) Table summarizing angiogenesis-, inflammation- and MEK/ERK-related pathways identified by GSEA analysis of LRRK2 G2019S vs. control iPSC-derived astrocytes. (B) Heatmap displaying genes enriched (yellow) in at least two angiogenesis-, inflammation-related pathways identified in the GSEA analysis. RNA-seq data were obtained from two (*Di Domenico et al.*), three (*de Rus Jacquet et al.*) or four (*Booth et al.*) biological replicates. Abbreviations: ES, Enrichment Score; NES, Normalized Enrichment Score; NOM p-val, Nominal p-value; FDR q-val, False Discovery Rate q-value. Source data are provided as a Source Data file.

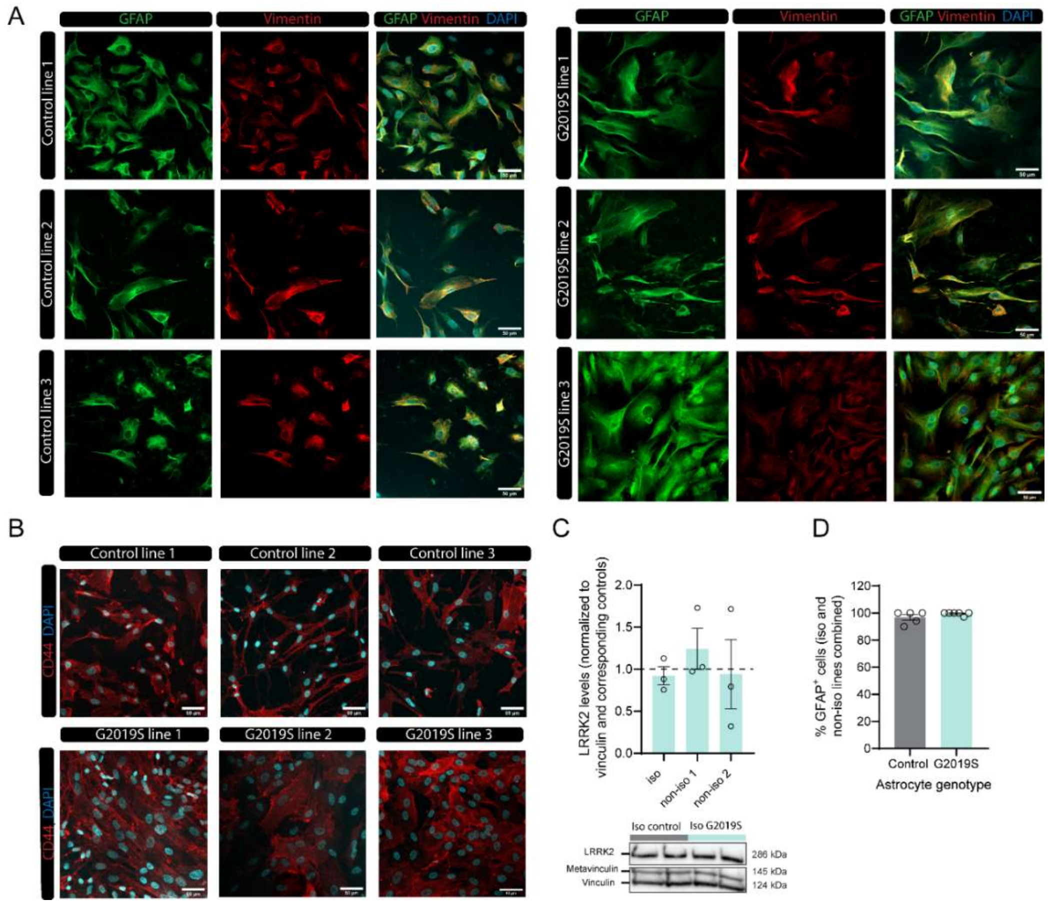

Supplementary Figure 3. Differentiation of iPSCs into astrocytes. (A-B) Representative confocal images of immunostained control and LRRK2 G2019S iPSC-derived astrocytes show expression of astrocyte markers GFAP (green), vimentin (red), merged GFAP (green) and vimentin (red) with the nuclear marker DAPI (blue) (A), and merged astrocyte marker CD44 (red) with DAPI (blue) (B). (C) Graph showing LRRK2 protein levels in iPSC-derived isogenic and non-isogenic control and LRRK2 G2019S astrocytes. LRRK2 levels are normalized to vinculin (housekeeping control), and the data is shown as the fold change LRRK2 value of mutant vs control astrocytes. Data is shown for all three iPSC lines, and the immunoblot is a representative image of LRRK2 and vinculin signal in the isogenic line. (D) Graph showing the proportion of GFAP-positive astrocytes in the iPSC-derived cultures. Data for all independent iPSC lines are combined into a single graph. Error bars represent mean + standard error of the mean (SEM). Statistical analysis was performed using one sample t-test with a theoretical mean of 1 (C), or two-tailed unpaired Student's t-test with equal standard deviation (s.d.) (D). Data are from three (C), four (D, control), or five (D, G2019S) independent biological replicates. Outliers were identified using Grubbs' test with an alpha value set at 0.05 and removed from analysis. Micrographs in (A-B) are representative of each astrocyte line. Control and G2019S lines 1 and 2 had already been validated in a published article (de Rus Jacquet et al, eLife, doi: 10.7554/eLife.73062). Immunofluorescence experiments for control and G2019S lines 3 were performed as follows: n=1 (control line 1) and n=3 (G2019S line 3). Scale bars: 50 µm (A, B). Source data are provided as a Source Data file.

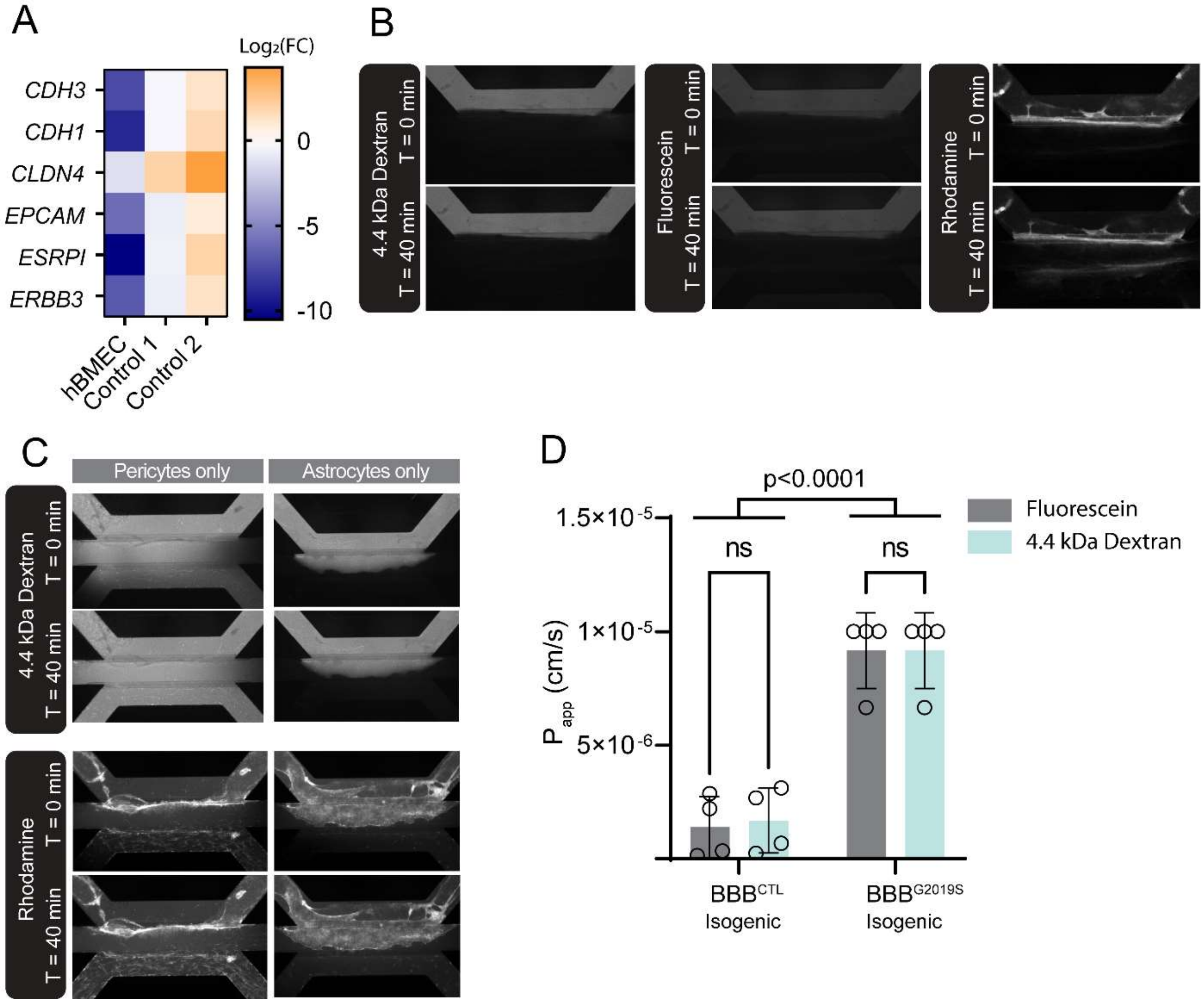

Supplementary Figure 4. Complementary BBB-chip validation experiments. (A) Gene expression analysis by qPCR of markers of epithelial cells identity in primary human BMECs (hBMEC) or BMEC-like cell monolayers produced from two different control iPSC lines (named *Control 1* and *Control 2*). The heatmap represents the $\log_2$(fold change) values of hBMEC or BMEC-like cells vs. undifferentiated iPSCs. (B-C) Representative images of barrier integrity assays using 4.4 kDa dextran-TMRE, rhodamine, or fluorescein in BBB^{CTL} (B), or in BBB-chips produced with control BMEC-like cells in the vascular compartment and either pericytes or astrocytes (i.e. a single glial type) in the brain compartment (C). (D) Quantification of fluorescein and 4.4 kDa dextran-TMRE apparent permability (P_{aap}) values in BBB^{G2019S} and BBB^{CTL} chips. Data was collected from three (A) or four (D) biological replicates; error bars represent mean + SEM. Statistical analysis in (D) was performed using a two-way ANOVA with Šídák's multiple comparisons test. The BBB^{CTL} and BBB^{G2019S} nomenclature refers to the presence of either control or LRRK2 G2019S astrocytes in the brain compartment of the BBB-chips. Abbreviation: cm, centimeter; kDa, kilodalton; FC, fold change; ns, not significant; s, seconds. Source data are provided as a Source Data file.

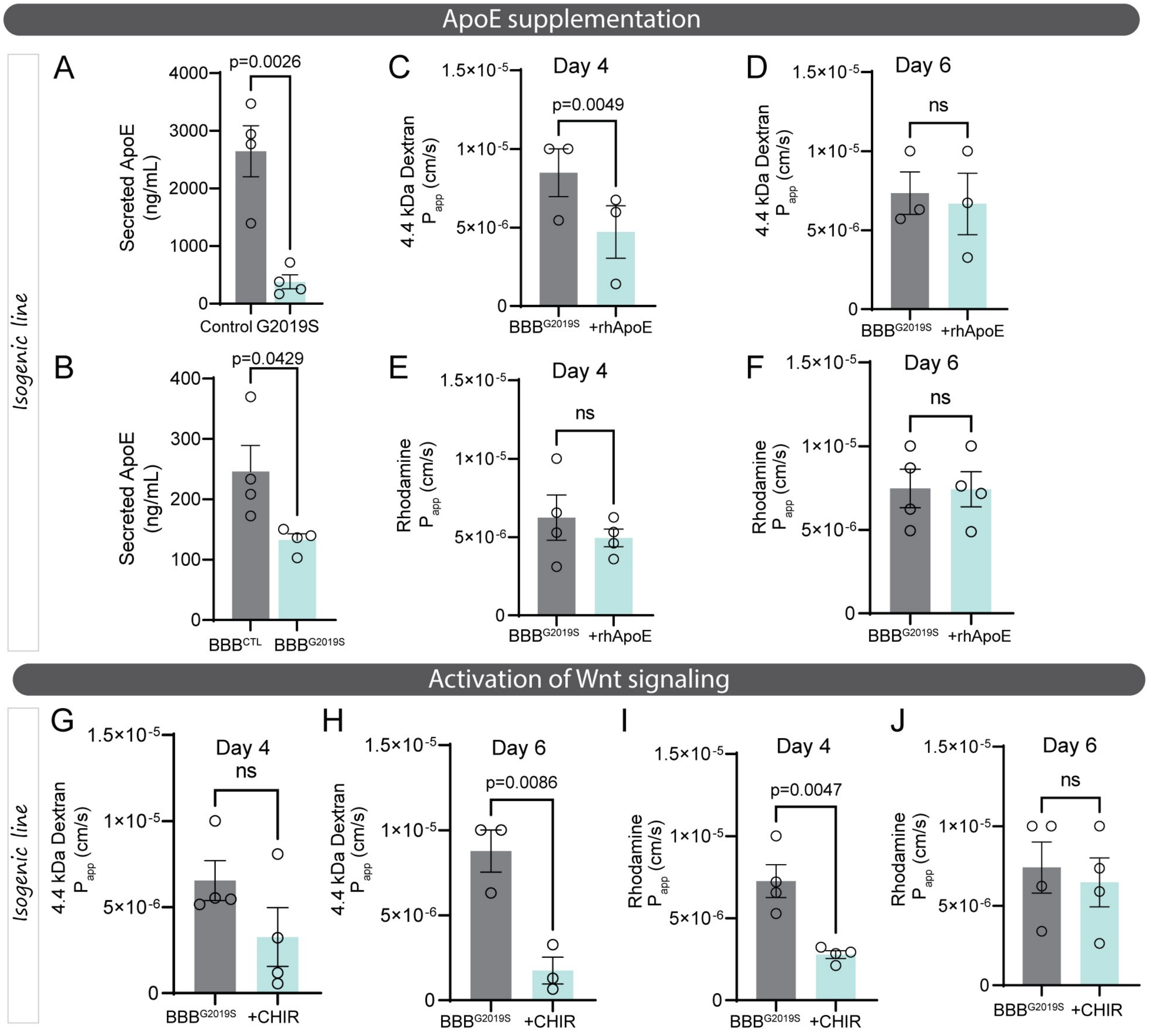

Supplementary Figure 5. Activation of angiogenic signals is not sufficient to sustain barrier integrity in the BBB-chip. (A-B) ELISA-based quantification of ApoE in astrocyte conditioned media collected from monolayer cultures (A), or in glia conditioned media collected from the brain compartment of BBB^{CTL} and BBB^{G2019S} chips (B). (C) Graphs showing vessel permeability to 4.4 kDa dextran-TMRE (C, D) and rhodamine (E, F) when the brain compartment of a BBB^{G2019S} chip is supplemented with recombinant human ApoE (rhApoE) for the duration of the experiment. (G-J) Graphs showing vessel permeability to 4.4 kDa dextran-TMRE (G, H) and rhodamine (I, J) when the vessel compartment of a BBB^{G2019S} chip is supplemented with CHIR99021, a small molecule activator of the pro-angiogenesis Wnt signaling pathway, for the duration of the experiment. These experiments were performed using the isogenic iPSC pair. Data are from three (A, C-D, H) or four (B, E-G, I-J) biological replicates; error bars represent mean + SEM. Statistical analysis was performed using two-tailed unpaired Student's t-test with equal s.d.. The BBB^{G2019S} nomenclature refers to the presence of LRRK2 G2019S astrocytes in the brain compartment of the BBB-chips. Abbreviation: cm, centimeter; ns, not significant; P_{app}, apparent permeability; s, seconds. Source data are provided as a Source Data file.

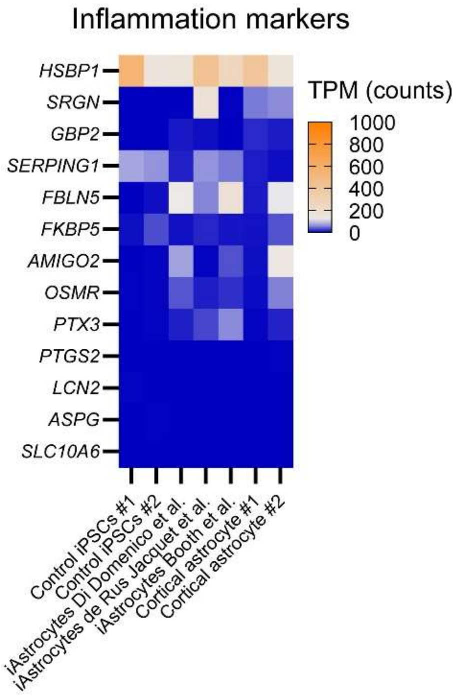

Supplementary Figure 6. Control astrocytes do not express genes associated with a reactive state. Heatmap showing expression levels of inflammatory markers in all datasets used for the RNA-seq analysis. The data labeled "iAstrocytes de Rus Jacquet et al." is representative of astrocytes produced for this study because the same iPSC lines and astrocyte differentiation protocol were used. For comparison, the data also includes expression levels in iPSCs and human primary cortical astrocytes provided with these datasets. Abbreviation: TPM, transcripts per million. Source data are provided as a Source Data file.

**Sex**	**Number of individual lines**	**Age of onset**	**Age of donor**	**Ethnicity**	**Source tissue**	**Isogenic?**	**Reprogramming methods**
iPSC lines included in the meta-analysis (Figure 1)							
**Di Domenico et al.**							
M, control	SP09	-	66	Caucasian	Fibroblasts	No	Retroviral vectors
F, PD	SP12	50	63	Caucasian	Fibroblasts	No	Retroviral vectors
**Booth et al.**							
F, control	1 (NHDF1)	-	44	N/A	Fibroblasts	No	Retroviral vector
M, control	1 (JR053-6)	-	68	N/A	Fibroblasts	No	Retroviral vector
F, control	1 (SFC840-03-03)	-	67	N/A	Fibroblasts	No	Sendai vector
M, PD	1 (JR036-1)	N/A	49	N/A	Fibroblasts	No (sibling of MK144-7)	Retroviral vector
F, PD	1 (MK144-7)	N/A	57	N/A	Fibroblasts	No, (sibling of JR036-1)	Retroviral vector
M, PD	1 (SFC855-03-06)	N/A	57	N/A	Fibroblasts	No	Sendai vector
F, PD	1 (SFC832-03-19)	N/A	77	N/A	Fibroblasts	No	Sendai vector
**De Rus Jacquet et al.**							
F, gene corrected	1 line (IM2-GC)	-	-	N/A	Fibroblasts	Yes	Retroviral vector, reprogrammed by [16]
F, PD	1 line (IM2)	N/A	-	N/A	Fibroblasts	Yes	Retroviral vector, reprogrammed by [16]
iPSC lines used for biological experiments in this paper							
F, gene corrected	1 line (IM2-GC)	-	-	N/A	Fibroblasts	Yes	Retroviral vector, reprogrammed by [16]
F, PD	1 line (IM2)	N/A	-	N/A	Fibroblasts	Yes	Retroviral vector, reprogrammed by [16]

F, control	1 line (36091)	-	63	Caucasian	Fibroblast	No	Episomal vectors
F, PD	1 line (33879)	N/A	66	Caucasian	Fibroblast	No	Episomal vectors
F, control	1 line (38554)	-	39	Caucasian	Fibroblast	No	Retroviral vectors
F, PD	1 line (3876)	N/A	60	Not Hispanic or Latino	Fibroblast	No	Retroviral vectors

Supplementary table 1. List of iPSC lines used in this study.

REAGENT or RESOURCE	SOURCE	IDENTIFIER
Cell culture reagents		
Accutase	Sigma-Aldrich	A6964
Collagen I	Corning	354249
Collagen IV	Sigma-Aldrich	C5333
E8 medium	StemCell Technologies	05990
Fibronectin	Sigma-Aldrich	F0895
Geltrex LDEV-Free, hESC-Qualified, Reduced Growth Factor Basement Membrane Matrix	Thermo Fisher Scientific	A1413301
Human endothelial serum-free medium	Thermo Fisher Scientific	11111044
mTeSR Plus medium	StemCell Technologies	100-0276
OrganoPlate 3-lane 40	Mimetas	4004-400-B
Platelet-poor human plasma	Sigma	2918
Pericyte medium	ScienCell Research Laboratories	1201
ReLeSR	StemCell Technologies	05872
Transwell polycarbonate inserts	Millipore Sigma	CLS3415-48EA
Antibodies		
anti-AKT1/2/3	Santa Cruz Biotechnology	sc8312
anti-Alpha-smooth muscle actin	Sigma	C6198
anti-CD31	eBiosciences	14-0311-81
anti-claudin 5	Thermo Fisher Scientific	35-2500
anti-GAPDH	Cell Signaling Technologies	2118
anti-GAPDH	Applied Biological Materials	G041
anti-GFAP	BD Biosciences	556328
anti-GLUT1	Novus Biologicals	NBP2-75785
anti-p38 MAPK	Cell Signaling Technologies	8690
anti-p44/42 MAPK	Cell Signaling Technologies	4695
anti-occludin	Invitrogen	71-1500
anti-phospho-AKT (Ser473)	Cell Signaling Technologies	9271
anti-phospho-p38 MAPK (Thr180/Tyr182)	Cell Signaling Technologies	4511
anti-phospho-p44/42 MAPK (Thr202/Tyr204)	Cell Signaling Technologies	4370
anti-VE-cadherin (for IF)	R&D Systems	AF938
anti-VE-cadherin (for western blot)	Abcam	ab33168
anti-ZO1	Invitrogen	61-7300
anti-laminin	Dako Agilent	Z0097
anti-tyrosine hydroxylase	Millipore Sigma	MAB318
Goat anti-mouse Alexa Fluor 488	Invitrogen	A11029
Donkey anti-mouse Alexa Fluor 555	Invitrogen	A31570
Goat anti-rabbit Alexa Fluor 488	Invitrogen	A11034

Donkey anti-rabbit Alexa Fluor 555	Invitrogen	A31572
Donkey anti-rabbit Alexa Fluor 546	Invitrogen	A10040
Donkey anti-goat Alexa Fluor 488	Invitrogen	A11055
HRP-goat anti-rabbit	Jackson ImmunoResearch	111-035-144
HRP-goat anti-mouse	Jackson ImmunoResearch	115-035-166
Chemicals, Peptides, and Recombinant Proteins		
Clarity Western ECL Substrate	Bio-Rad Laboratories	1705060
Clarity Max Western ECL Substrate	Bio-Rad Laboratories	1705062s
Dextran-TMRE	Millipore Sigma	T1037
FGFb	StemCell Technologies	78003.1
Fluorescein	Thermo Fisher Scientific	F1300
Halt protease and phosphatase inhibitor cocktail	Thermo Fisher Scientific	78443
Immobilon Forte Western HRP substrate	Millipore	WBLUF0500
PD0325901	Cayman Chemical Company	13034
Recombinant Human IL-8 (CXCL8)	PeproTech	200-08M
Recombinant Human IL-6	PeproTech	200-06
Retinoic acid	Sigma	2625
Rhodamine B	Millipore Sigma	83689
1 X RIPA buffer	Cell Signaling Technologies	9806
SCH772984	Cayman Chemical Company	19166
SuperBlock	Thermo Fisher Scientific	37515
TRIzol	Thermo Fisher Scientific	15596026
Y-27632	StemCell Technologies	72302
Critical Commercial Assays		
BCA assay	Thermo Fisher Scientific	A53226
Direct-zol RNA Miniprep kit	Zymo Research	R2051
IL-6 ELISA (microfluidic plate)	R&D Systems	DY206-05
IL-6 ELISA (monolayers)	Thermo Fisher Scientific	KHC0061
IL-8 ELISA (microfluidic plate)	R&D Systems	DY208-05
IL-8 ELISA (monolayers)	Thermo Fisher Scientific	KAC1301
KAPA SYBR FAST master mix	Roche	07959397001
Proteome Profiler Human Angiogenesis Array Kit	R&D Systems	ARY007
Proteome Profiler Human Cytokine Array	R&D Systems	ARY005B
RevertAid First Strand cDNA Synthesis Kit	Thermo Fisher Scientific	K1622
RNeasy kit	Qiagen	74104
4-20% Tris-Glycine gel	Thermo Fisher Scientific	XP04200BOX
Tris-Glycine SDS running buffer	Thermo Fisher Scientific	LC2675

Experimental Models: Cell Lines		
Isogenic LRRK2 G2019S iPSCs	by Prof. Dr. Thomas Gasser (Universitätsklinikum Tübingen) and Prof. Dr. Hans R. Schöler (Max-Planck Institute)	(Reinhardt *et al.*, 2013)
Non-isogenic LRRK2 G2019S iPSCs	Dr. Randall T. Moon (Howard Hughes Medical Institute/University of Washington)	(de Rus Jacquet, 2021)
Non-isogenic LRRK2 G2019S iPSCs	NINDS repository	ND40018, ND38554.
Primary human brain vascular pericytes	ScienCell Research Laboratories	1200
Software and Algorithms		
Beacon Designer Lite 8.16	Premier Biosoft	
ComplexHeatmap v2.6.2	Bioconductor	
Database for Annotation, Visualization, and Integrated Discovery v6.8	https://david.ncifcrf.gov	
DESeq2 v1.30.1	Bioconductor	(Love et al., 2014)
FactoMineR v2.4	https://cran.r-project.org/	(Lê, 2008)
FastQC v0.11.8	GitHub	(Andrews, 2010)
GraphPad Prism version 8.0	https://www.graphpad.com/scientific-software/prism/	
ggplot2 v3.3.3	https://cran.r-project.org/	(Wickham, 2011)
Kallisto v0.46.2	GitHub	(Bray et al., 2016)
MultiQC v1.8	GitHub	(Ewels et al., 2016)
R v4.0.3	https://www.r-project.org/	
RUVSeq v1.24.0	Bioconductor	(Risso et al., 2014)
Tidyverse v2.0.0	https://cran.r-project.org/	(Wickham et al., 2019)
OTHER		
Polyvinylidene fluoride (PVDF) transfer membrane	Thermo Fisher Scientific	88518
Previously published RNA-seq dataset	(di Domenico et al., 2019)	GEO accession number GSE116124
Previously published RNA-seq dataset	(de Rus Jacquet, 2021)	GEO accession number GSE152768
Previously published RNA-seq dataset	(Booth et al., 2019)	GEO accession number GSE120306

Supplementary Table 2. Resources Table

**Gene**	**Forward Sequence**	**Reverse Sequence**	**Species**	**Source**
ABCB1	TACCATACAGAAACTCTTT	CTAACAGAACATCCTCAA	Human	This study
AMIGO2	TTCTGGATTCTGAGTGGATTC	TGCTGGTGATGTTGTTATGA	Human	This study
ASPG	CCTCTTCGACTCCAGTGACAT	TGCTCGTAGTGCCTCTTGAT	Human	This study
CDH1	CCTGGTTCAGATCAAATC	GTGAGAGAAGAGAGTGTA	Human	This study
CDH3	ATAATGATGACTTCACTGT	TTAGACTTGAGCTGATTC	Human	This study
CDH5	TATGAGATCGTGGTGGAA	TACTTGGTCTGGGTGAAG	Human	This study
CLDN4	TTCCAAGGACACTAATGA	AAACAGAAACCACAAAGA	Human	This study
CLDN5	TTAACAGACGGAATGAAGT	GAAGCGAAATCCTCAGTC	Human	This study
CXCL8	GACCACACTGCGCCAACAC	CTTCTCCACAACCCTCTGCAC	Human	doi.org/10.1038/nbt1288
EPCAM	AATTGTTGTGCTGGTTAT	TTGCTATTTCCCTTCTTC	Human	This study
ERBB3	TACTACCTTGAGGAACAT	CTTAATTGGAGTCTTGGC	Human	This study
ESRP1	TCCAAGAAGAATGTACTATT	TGTCAATATCAGGTGAAC	Human	This study
FBLN5	CCAGTGCTTAGACATTGA	AGACCTTCCATCCTCATT	Human	This study
FKBP5	TGAAGATGGAGGCATTATC	AGGTGGATTTCTACTGTTG	Human	This study
GBP2	GGACCAACTTCACTATGT	TGAGTCGTCTACAGAATTG	Human	This study
GLUD1	ATGTGAGTGTAGATGAAGTA	TCATTATCAGTATAGTTCTTGG	Human	This study
HSBP1	GAGTTGAAGGTTGCTAAT	CCAAGATGTGAAGATACC	Human	This study
ICAM1	ATGCCCAGACATCTGTGTCC	GGGGTCTCTATGCCCAACAA	Human	doi.org/10.3892/mmr.2019.10512
IL6	GGATTCAATGAGGAGACTT	ATCTGTTCTGGAGGTACT	Human	This study
KDR	AGGAATCAGTCAGTATCT	ACTTCTGGTTCTTCTAAC	Human	This study
LCN2	GGCAACATTAAGAGTTACC	TTGAAGAACACCATAGCA	Human	This study
NLRP3	GCTTCAGGTGTTGGAATTAG	GAGGTCAGAAGTGTGGAA	Human	This study
OCLN	AATATCCACCTATCACTTCAGA	CAGCAGCCATGTACTCTT	Human	This study
OSMR	TACCAGAGTGAAGTCTTG	GATGATAAGGAAGGTTGTG	Human	This study
PECAM1	AGTCATTACGGTCACAAT	CTGAGGACACTTGAACTT	Human	This study
PTGS2	AGGCTAATACTGATAGGAGAG	ATTCAGCAGCAATACGATT	Human	This study
PTX3	TGCAGTGTTGGCCGAGAAC	GGCGTGGGGTCCTCAGTG	Human	This study
SERPING1	GCTATCTACCTGAGTGCCAAGT	TTTGAAGTGAAAGGGTTCCATTCT	Human	This study
SLC10A6	TCAGAACATAGGAATTACC	CCCAATCTTGAGAATGAT	Human	This study
SLC2A1	CTGAGCATCATCTTCATC	TTCTTTAGCACACTCTTG	Human	This study
SOX17	GGACATGAAGGTGAAGG	GACTTGCCCAGCATCTT	Human	This study
SOX9	GCTCTGGAGACTTCTGAA	GGCTGGTACTTGTAATCC	Human	This study
SRGN	CTCAGTTCAAGGTTATCC	TTTCTTCAAGGCAGTTTG	Human	This study
TEK	TGCTACTTAACAACTTACA	GAGGAAGAATGTCACTAA	Human	This study
TJP1	GATGTTGTATTGAAGATAAATGG	TCTGAAATGTCGTCTCTC	Human	This study
VCAM1	TGATGTTCAAGGAAGAGA	TGGCAGGTATTATTAAGGA	Human	This study
VWF	CCTGATTTCTGTGCTATG	TCCAACATGACTTTATCTG	Human	This study

Supplementary Table 3. List of RT-qPCR primers used in this study

UNCROPPED SCANS OF WESTERN BLOTS
for Supplementary Figures

Lane	Experimental conditions
1	Astrocyte (isogenic)-control
2	Astrocyte (isogenic)-control
3	Astrocyte (isogenic)-LRRK2 G2019S
4	Astrocyte (isogenic)-LRRK2 G2019S
5	Astrocyte (isogenic)-LRRK2 G2019S
6	Experimental condition unrelated to this manuscript
7	Astrocyte (non-isogenic pair 1)-control
8	Astrocyte (non-isogenic pair 1)-control
9	Astrocyte (non-isogenic pair 1)-control
10	Astrocyte (non-isogenic pair 1)-control
11	Astrocyte (non-isogenic pair 1)-LRRK2 G2019S
12	Astrocyte (non-isogenic pair 1)-LRRK2 G2019S
13	Astrocyte (non-isogenic pair 2)-control
14	Astrocyte (non-isogenic pair 2)-control

Bands selected for display in Supplementary Figure 3: lanes 1-4

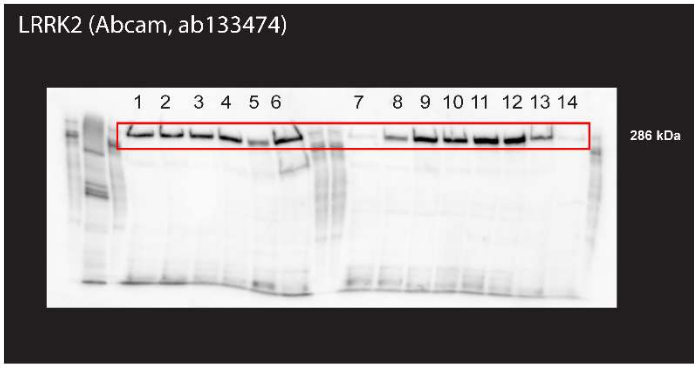

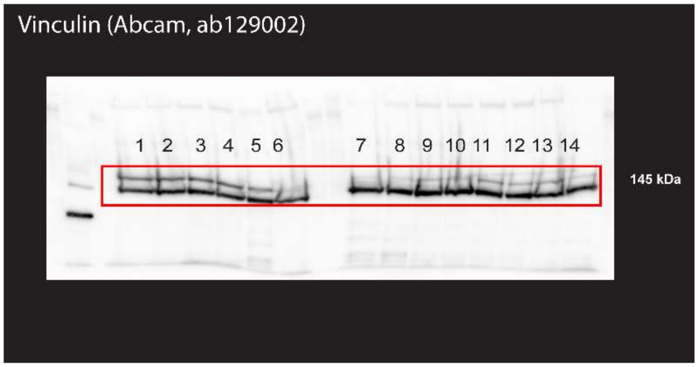

Lane	Experimental conditions
1	Astrocyte (non-isogenic pair 2)-LRRK2 G2019S
2	Experimental condition unrelated to this figure
3	Experimental condition unrelated to this figure
4	Astrocyte (non-isogenic pair 2)-LRRK2 G2019S
5	Experimental condition unrelated to this figure
6	Experimental condition unrelated to this figure
7	Astrocyte (non-isogenic pair 2)-LRRK2 G2019S
8	Experimental condition unrelated to this figure
9	Experimental condition unrelated to this figure
10	Astrocyte (non-isogenic pair 2)-control
11	Astrocyte (non-isogenic pair 2)-control

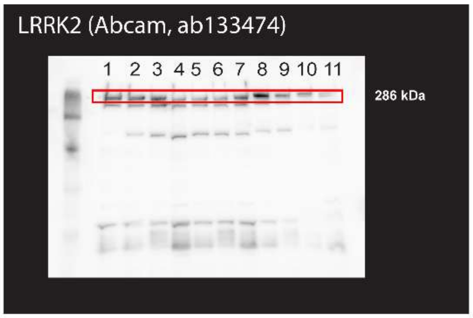

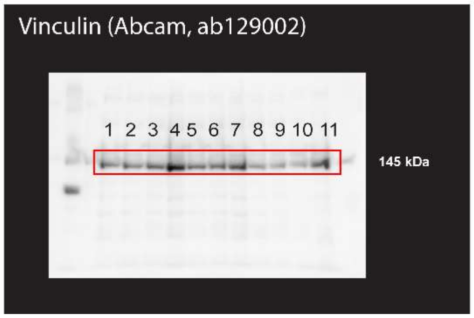

Lane	Experimental condition
1	Astrocyte (non-isogenic pair 1)-control
2	Astrocyte (non-isogenic pair 1)-control
3	Astrocyte (non-isogenic pair 1)-control
4	Astrocyte (non-isogenic pair 1)-G2019S
5	Astrocyte (non-isogenic pair 1)-G2019S
6	Experimental condition unrelated to this manuscript

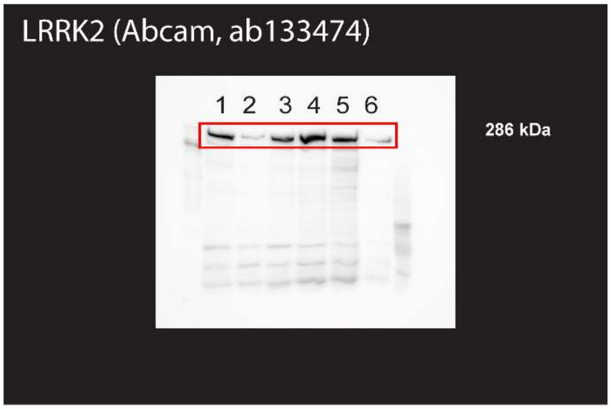

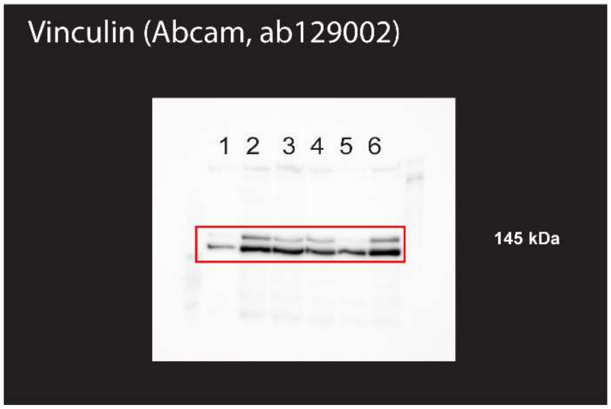

These images refer to Supplementary Figure 3C.
