# Supplementary material for: The contribution of inflammatory astrocytes to BBB impairments in a brain-chip model of Parkinson’s disease
Source: Nat Commun. 2023 Jun 20;14:3651. doi: 10.1038/s41467-023-39038-8 (PMC10282096; doi:10.1038/s41467-023-39038-8)
Supplement: Supplementary file 3 — Description of Additional Supplementary Files [file 41467_2023_39038_MOESM3_ESM.pdf]

## Description of Additional Supplementary Files

**Supplementary Data 1**. RNA-sequencing meta-analysis. Data is compiled from 3 datasets: de Rus Jacquet et al., Booth et al. and di Domenico et al.. The tabs "All genes_Downregulated" and "All genes_Upregulated" show the combined dysregulated genes for these 3 datasets (duplicates removed) and compares LRRK2 G2019S vs control iPSC-derived astrocytes. Gene Ontology tabs: sub-categories are extracted from DAVID Gene Ontology analysis. The fold change value of LRRK2 G2019S vs. control astrocytes are provided in this supplementary file, and these are the data used to generate the heatmaps provided in Figure 1. The genes that are dysregulated in at least 2 datasets were used to generate the histograms provided in Figure 1.
