# Supplementary material for: The contribution of inflammatory astrocytes to BBB impairments in a brain-chip model of Parkinson’s disease
Source: Nat Commun. 2023 Jun 20;14:3651. doi: 10.1038/s41467-023-39038-8 (PMC10282096; doi:10.1038/s41467-023-39038-8)
Supplement: Supplementary file 5 — Reporting Summary [file 41467_2023_39038_MOESM5_ESM.pdf]

nature portfolio

Corresponding author(s): Aurelie de Rus Jacquet, Francesca Cicchetti

Last updated by author(s): May 24, 2023

Reporting Summary

Nature Portfolio wishes to improve the reproducibility of the work that we publish. This form provides structure for consistency and transparency in reporting. For further information on Nature Portfolio policies, see our Editorial Policies and the Editorial Policy Checklist.

Statistics

For all statistical analyses, confirm that the following items are present in the figure legend, table legend, main text, or Methods section.

n/a	Confirmed	
☐	☒	The exact sample size (*n*) for each experimental group/condition, given as a discrete number and unit of measurement
☐	☒	A statement on whether measurements were taken from distinct samples or whether the same sample was measured repeatedly
☐	☒	The statistical test(s) used AND whether they are one- or two-sided<br>*Only common tests should be described solely by name; describe more complex techniques in the Methods section.*
☒	☐	A description of all covariates tested
☐	☒	A description of any assumptions or corrections, such as tests of normality and adjustment for multiple comparisons
☐	☒	A full description of the statistical parameters including central tendency (e.g. means) or other basic estimates (e.g. regression coefficient) AND variation (e.g. standard deviation) or associated estimates of uncertainty (e.g. confidence intervals)
☐	☒	For null hypothesis testing, the test statistic (e.g. *F*, *t*, *r*) with confidence intervals, effect sizes, degrees of freedom and *P* value noted<br>*Give P values as exact values whenever suitable.*
☒	☐	For Bayesian analysis, information on the choice of priors and Markov chain Monte Carlo settings
☒	☐	For hierarchical and complex designs, identification of the appropriate level for tests and full reporting of outcomes
☒	☐	Estimates of effect sizes (e.g. Cohen's *d*, Pearson's *r*), indicating how they were calculated

Our web collection on statistics for biologists contains articles on many of the points above.

Software and code

Policy information about availability of computer code

Data collection	Data were collected using GEN5 version 2.09, confocal images were collected using ZEN version 2.3.
Data analysis	RNA-seq data was analyzed using the following R packages: fastp v0.20.0 , FastQC v0.11.8, MultiQC v1.8 , Kallisto v0.46.2, FactoMineR v2.4, ggplot2 v3.3.3, RUVSeq v1.24.0, DESeq2 v1.30.1, tidyverse v2.0.0, and ComplexHeatmap v2.6.2. All R analysis were done in R v4.0.3. Gene ontology enrichment analysis was performed using the Database for Annotation, Visualization, and Integrated Discovery (DAVID v6.8, https://david.ncifcrf.gov/). GSEA analysis was performed using the broad institute GSEA-P tools. Other data were analyzed using GraphPad Prism version 8.0 (La Jolla, CA), ImageJ version 1.51n and 1.53T, myImageAnalysis Software version 1.1.<br><br>Custom code generated to analyze the RNA-seq data is available at: https://github.com/ArnaudDroitLab/de_rus_jacquet_2023

For manuscripts utilizing custom algorithms or software that are central to the research but not yet described in published literature, software must be made available to editors and reviewers. We strongly encourage code deposition in a community repository (e.g. GitHub). See the Nature Portfolio guidelines for submitting code & software for further information.

Data

Policy information about availability of data

All manuscripts must include a data availability statement. This statement should provide the following information, where applicable:

- Accession codes, unique identifiers, or web links for publicly available datasets
- A description of any restrictions on data availability
- For clinical datasets or third party data, please ensure that the statement adheres to our policy

RNA-seq datasets analyzed in this study were previously published and are publicly available, and they were obtained from GEO repository using accession numbers GSE116124 (https://www.ncbi.nlm.nih.gov/geo/query/acc.cgi?acc=GSE116124), GSE152768 (https://www.ncbi.nlm.nih.gov/geo/query/acc.cgi?acc=GSE152768), GSE120306 (https://www.ncbi.nlm.nih.gov/geo/query/acc.cgi?acc=GSE120306).

This study also accessed the following databases: Ensembl release 100 (https://www.ensembl.org/info/website/archives/index.html?redirect=no), the Human Transcription Factors database (http://humantfs.ccbr.utoronto.ca/), the HumanTFDB portal (http://bioinfo.life.hust.edu.cn/AnimalTFDB/), the TF2DNA database (https://www.fiserlab.org/tf2dna_db/), QuickGO (https://www.ebi.ac.uk/QuickGO/), GSEA (https://www.gsea-msigdb.org/gsea/index.jsp), Biocarta (https://maayanlab.cloud/Harmonizome/dataset/Biocarta+Pathways), KEGG (https://www.genome.jp/kegg/).

Research involving human participants, their data, or biological material

Policy information about studies with human participants or human data. See also policy information about sex, gender (identity/presentation), and sexual orientation and race, ethnicity and racism.

Reporting on sex and gender	Sex and gender were not considered as a variable in this study, however the iPSC lines used were generated from female donors. In addition, the postmortem tissue used in this work originated from donors of both sex. The postmortem work presented in Figure 8 is a small-scale study of 5 control and 5 PD donors, therefore we thought it would be best to show aggregated data into a single graph instead of disaggregating the data by sex. The abstract reflects the use of female iPSCs and both male/female human postmortem tissue. Table 1 and Supplementary Table 1 list the demographic information for the postmortem tissue and iPSC lines, respectively.
Reporting on race, ethnicity, or other socially relevant groupings	This study does not report on race, ethnicity or other social groupings.
Population characteristics	Population characteristic data is provided in Table 1 and include sex, disease status, age, postmortem interval and cause of death.
Recruitment	Postmortem brain tissue used in Figure 8 was obtained from the CERVO brain bank in Quebec city, Canada.
Ethics oversight	Ethical approval for the use of human tissue was received from Laval University's and CRCHUQ-UL institutional review board (i.e. CER), projet ID 2012-1138, A13-02-1138, SIRUL 107683.

Note that full information on the approval of the study protocol must also be provided in the manuscript.

Field-specific reporting

Please select the one below that is the best fit for your research. If you are not sure, read the appropriate sections before making your selection.

[x] Life sciences [ ] Behavioural & social sciences [ ] Ecological, evolutionary & environmental sciences

For a reference copy of the document with all sections, see nature.com/documents/nr-reporting-summary-flat.pdf

Life sciences study design

All studies must disclose on these points even when the disclosure is negative.

Sample size	In vitro experiments:<br>A minimum of three biological replicates were independently collected, and the details of the number of replicates and sample size are provided in the figure legends. No statistical methods were used to predetermine the number of biological replicates and sample size. A minimum of three independant biological replicates were performed to evaluate reproducibility, as done in previous work (https://elifesciences.org/articles/73062). Data was collected using isogenic and non-isogenic iPSC pairs, and these pairs are explicitely identified in the figures and figure legends.<br><br>Human postmortem study:<br>Brain sections originating from 5 control and 5 persons with Parkinson's disease were used for this study. No statistical methods were used to predetermine the sample size, we established the sample size based on tissue availability.
Data exclusions	Outliers in were identified using Grubbs' test with an alpha value set at 0.05 and removed from analysis. This criteria is pre-established because of the inherent variability of the BBB-chip model. This information is included in the figure legends of the corrected data.

Replication	In vitro experiments were validated across a minimum of three independent biological replicates. Findings were replicated across several iPSC pairs generated from an independent donors. We did not observe discrepancies in the results collected.<br><br>Postmortem human experiments were performed on tissue donated by 5 different control and 5 different PD donors.
Randomization	For each cell type, iPSCs were differentiated into multiple independant batches, and cryopreserved for future use. Cells were then randomly assigned to the experimental groups.
Blinding	Experiments at-risk for biais (analysis of BBB-chips):<br>The investigators were blinded to group allocation during data analysis via the assignement of sample IDs (BBB-chips were assigned a Beacon ID at the time the images were taken, which de-identified the images).<br><br>Experiments at-risk for biais (human postmortem analysis):<br>The investigators were blinded to group allocation during data analysis via the use of sample IDs.<br><br>Other experiments were not evaluated using subjective measures, and the investigators were not blinded to group allocation.

Reporting for specific materials, systems and methods

We require information from authors about some types of materials, experimental systems and methods used in many studies. Here, indicate whether each material, system or method listed is relevant to your study. If you are not sure if a list item applies to your research, read the appropriate section before selecting a response.

Materials & experimental systems

n/a	Involved in the study
☐	☒ Antibodies
☐	☒ Eukaryotic cell lines
☒	☐ Palaeontology and archaeology
☒	☐ Animals and other organisms
☒	☐ Clinical data
☒	☐ Dual use research of concern
☒	☐ Plants

Methods

n/a	Involved in the study
☒	☐ ChIP-seq
☒	☐ Flow cytometry
☒	☐ MRI-based neuroimaging

Antibodies

Antibodies used	All antibody information is detailed in supplementary table 2 and below. Information about dilution for each antibody is provided in the manuscript.<br><br>Antibodies<br>anti-AKT1/2/3 Cell Signaling Technologies sc8312<br>anti-Alpha-smooth muscle actin Sigma C6198<br>anti-CD31 eBiosciences 14-0311-81<br>anti-claudin 5 Thermo Fisher Scientific 35-2500<br>anti-GAPDH Cell Signaling Technologies 2118<br>anti-GAPDH Applied Biological Materials G041<br>anti-GFAP BD Biosciences 556328<br>anti-GLUT1 Novus Biologicals NBP2-75785<br>anti-p38 MAPK Cell Signaling Technologies 8690<br>anti-p44/42 MAPK Cell Signaling Technologies 4695<br>anti-occludin Invitrogen 71-1500<br>anti-phospho-AKT (Ser473) Cell Signaling Technologies 9271<br>anti-phospho-p38 MAPK (Thr180/Tyr182) Cell Signaling Technologies 4511<br>anti-phospho-p44/42 MAPK (Thr202/Tyr204) Cell Signaling Technologies 4370<br>anti-VE-cadherin (for IF) R&D Systems AF938<br>anti-VE-cadherin (for western blot) Abcam ab33168<br>anti-ZO1 Invitrogen 61-7300<br>anti-laminin Dako Agilent Z0097<br>Anti-tyrosine hydroxylase Millipore Sigma MAB318<br>Goat anti-mouse Alexa Fluor 488 Invitrogen A11029<br>Donkey anti-mouse Alexa Fluor 555 Invitrogen A31570<br>Goat anti-rabbit Alexa Fluor 488 Invitrogen A11034<br>Donkey anti-rabbit Alexa Fluor 555 Invitrogen A31572<br>Donkey anti-rabbit Alexa Fluor 546 Invitrogen A10040<br>Donkey anti-goat Alexa Fluor 488 Invitrogen A11055

	HRP-goat anti-rabbit Jackson ImmunoResearch 111-035-144<br>HRP-goat anti-mouse Jackson ImmunoResearch 115-035-166
Validation	Antibodies were selected based on previous utilization in published studies and reactivity with human proteins, since all our experiments were performed using human cells or tissue. Furthermore, we relied on validation statements on the manufacturer's website and customer reviews. BenchSci served as an additional resource to select antibodies for our study. In addition, we validated antibodies in-house by verifying proper cellular localization (immunofluorescence) or molecular weight (western blot).<br><br>anti-AKT1/2/3 Santa Cruz Biotechnology sc8312 Used in 524 publications (source: BenchSci)<br>anti-Alpha-smooth muscle actin Sigma C6198 Used in 402 publications (source: BenchSci)<br>anti-CD31 eBiosciences 14-0311-81 Used in 168 publications (source: BenchSci)<br>anti-claudin 5 Thermo Fisher Scientific 35-2500 Used in 206 publications (source: BenchSci)<br>anti-GAPDH Cell Signaling Technologies 2118 Used in 2.5K publications (source: BenchSci)<br>anti-GAPDH Applied Biological Materials G041 Used in 12 publications (source: BenchSci)<br>anti-GFAP BD Biosciences 556328 Used in 15 publications (source: BenchSci)<br>anti-GLUT1 Novus Biologicals NBP2-75785 Validation provided on the Manufacturer's website<br>anti-p38 MAPK Cell Signaling Technologies 8690 Used in 763 publications (source: BenchSci)<br>anti-p44/42 MAPK Cell Signaling Technologies 4695 Used in 2.1K publications (source: BenchSci)<br>anti-occludin Invitrogen 71-1500 Used in 383 publications (source: BenchSci)<br>anti-phospho-AKT (Ser473) Cell Signaling Technologies 9271 Used in 3.2K publications (source: BenchSci)<br>anti-phospho-p38 MAPK (Thr180/Tyr182) Cell Signaling Technologies 4511 Used in 766 publications (source: BenchSci)<br>anti-phospho-p44/42 MAPK (Thr202/Tyr204) Cell Signaling Technologies 4370 Used in 2K publications (source: BenchSci)<br>anti-VE-cadherin (for IF) R&D Systems AF938 Used in 20 publications (source: BenchSci)<br>anti-VE-cadherin (for western blot) Abcam ab33168 Used in 271 publications (source: BenchSci)<br>anti-ZO1 Invitrogen 61-7300 Used in 843 publications (source: BenchSci)<br>anti-laminin Dako Agilent Z0097 Used in 80 publications (source: BenchSci)<br>anti-tyrosine hydroxylase Millipore Sigma MAB318 Used in 326 publications (source: BenchSci)

Eukaryotic cell lines

Policy information about cell lines and Sex and Gender in Research

Cell line source(s)	Isogenic LRRK2 G2019S iPSCs provided by Prof. Dr. Thomas Gasser (Universitätsklinikum Tübingen) and Prof. Dr. Hans R. Schöler (Max-Planck Institute) (Reinhardt et al., 2013)<br><br>Non-isogenic LRRK2 G2019S iPSCs provided by Dr. Randall T. Moon (Howard Hughes Medical Institute/University of Washington) (de Rus Jacquet, 2021)<br><br>Non-isogenic LRRK2 G2019S iPSCs provided by the NINDS repository ND40018, ND38554.<br>Primary human brain vascular pericytes purchased at ScienCell Research Laboratories (catalog number 1200)
Authentication	LRRK2 G2019S mutants were genotyped as described in de Rus Jacquet et al. eLife 10:e73062
Mycoplasma contamination	Lines originating from the Coriell repository were controlled for absence of mycoplasma contamination.
Commonly misidentified lines<br>(See ICLAC register)	This study did not use commonly misidentified lines.
